# Evaporation kinetics of surfactant solution droplets on rice (*Oryza sativa*) leaves

**DOI:** 10.1371/journal.pone.0176870

**Published:** 2017-05-04

**Authors:** Zhao-Lu Zhou, Chong Cao, Li-Dong Cao, Li Zheng, Jun Xu, Feng-Min Li, Qi-Liang Huang

**Affiliations:** Key Laboratory of Integrated Pest Management in Crops, Ministry of Agriculture, Institute of Plant Protection, Chinese Academy of Agricultural Sciences, Beijing, China; Queensland University of Technology, AUSTRALIA

## Abstract

The dynamics of evaporating sessile droplets on hydrophilic or hydrophobic surfaces is widely studied, and many models for these processes have been developed based on experimental evidence. However, few research has been explored on the evaporation of sessile droplets of surfactant or pesticide solutions on target crop leaves. Thus, in this paper the impact of surfactant concentrations on contact angle, contact diameter, droplet height, and evolution of the droplets’ evaporative volume on rice leaf surfaces have been investigated. The results indicate that the evaporation kinetics of surfactant droplets on rice leaves were influenced by both the surfactant concentrations and the hydrophobicity of rice leaf surfaces. When the surfactant concentration is lower than the surfactant CMC (critical micelle concentration), the droplet evaporation time is much longer than that of the high surfactant concentration. This is due to the longer existence time of a narrow wedge region under the lower surfactant concentration, and such narrow wedge region further restricts the droplet evaporation. Besides, our experimental data are shown to roughly collapse onto theoretical curves based on the model presented by Popov. This study could supply theoretical data on the evaporation of the adjuvant or pesticide droplets for practical applications in agriculture.

## Introduction

Rice is one of the most widely grown crops in the world [1], and severely affected by plant diseases, insect pests, and weeds, all of which are mainly controlled by foliar spraying of pesticide solutions. Spray application is a complex dynamic process that involves many interdependent components. Transportation of an active ingredient is a complicated process, which starts with the preparation of the spray solution followed by atomization, translocation, and impact between the solution and the leaf surface [2]. The leaf surface characteristics, spray solution properties, and environmental conditions affect the spray deposition of pesticide solutions onto the target crop leaves. Thereby, the evaporation behavior of droplets significantly affects the deposition formation, drying process, penetration and translocation within the target crop tissue, and bio-efficiency. The high temperatures of rice paddies cause the rapid evaporation of pesticide droplets after spray, especially the rice leave surfaces which are hydrophobic [3] and highly water repellent, making it easy for the pesticide droplets to bounce and roll off. All of these factors could reduce the efficacy of pesticides and even have an influence on food quality, eco-environmental security, and human health [4]. Thus, the evaporation of pesticide droplets and their retention on rice leaf surfaces play important roles in effective control of pesticides. For example, the evaporation time is crucial for the efficiency of systemic pesticides, since they would be effective only after being absorbed by the plant. Longer evaporation times could endow plants more chance to contact with the active ingredient that helps control the disease.

The evaporation of sessile droplets on a solid substrate has been investigated both theoretically and experimentally [5–10]. Many studies have been reported in different research areas including surface patterning [11], inkjet printing [12], colloidal photonic crystal fabrication [13]. The evaporation of droplets is influenced by many factors, such as the chemical heterogeneity [14] and physical properties (e.g., the roughness of the substrate [15]), and the properties of the liquid droplet [16] of the substrate. Picknett and Bexon [17] studied the evaporation of sessile droplets in an open atmosphere and have developed two modes for droplets evaporation that act on droplets on a smooth substrate: the constant contact radius (CCR) mode and the constant contact angle (CCA) mode. Stauber *et al*. [18] reported a complete description of the relationship between the lifetime of a droplet on a solid substrate in a stick-slide mode and that of initially identical droplets in the extreme modes (CR and CA modes). They found that generally, the lifetime of a droplet is not constrained by the lifetimes of extreme modes. Also, if droplets evaporate in an idealized stick-slide mode the dependence of the lifetime on the initial contact angle is qualitatively different from that when the relationship between the initial and the receding contact angles is not taken into account [19]. The evaporation flux on the surface of droplets was assumed to be constant in early models [6, 8, 9, 20]. However, Deegan [21]and Popov [22] argued that the evaporation flux along the droplet surface is nonuniform. Popov [22] reported an analytical solution to describe the rate of mass loss in terms of the entire range of contact angles. Gelderblom *et al*. [23] verified experimentally the validity of Popov’s “diffusion-limited” model [22] for droplet evaporation on a superhydrophobic surface with a fixed contact line. The “diffusion-limited” model is widely used among experiments for sessile droplets with small contact angles (< 90°) when it is assumed that the evaporation of droplets is quasi-steady and limited by the diffusion of vapor in the quiescent atmosphere above the droplet. To date, relatively few research has been done for hydrophobic [6, 24–26] or super-hydrophobic [22, 23, 25, 27, 28] surfaces. The extreme CCA and CCR evaporation modes become indistinguishable on strongly hydrophobic substrates [29]. It is appropriate to use the so-called “2/3 power law” to extrapolate the lifetime of droplets that evaporate from strongly hydrophobic substrates for both CCR and CCA modes. The “diffusion-limited” model provides the good agreement with a smooth hydrophobic surface (contact angle ~120°), while the structured super-hydrophobic surface (contact angle ~160°) demonstrates a significant deviation from the diffusion-limited model [30, 31]. Experimental investigations of the evaporation kinetics of sessile water droplets on micropillared super-hydrophobic surfaces [32] have shown an initial large contact angle that provides a narrow wedge region. Within this region, the liquid−vapor diffusion is significantly restricted. Even fewer experiments have been performed for evaporation from disordered super-hydrophobic surfaces, such as natural real leaves. The wetting of natural leaf surfaces is different from that of artificial ones since the leaf roughness has an effect on the retention of water droplets on leaves [33]. The leaf surface roughness, related to the wettability [34], was effectively quantified by a fractal dimension analysis of cryo-scanning electron microscopy (cryo-SEM) [35]. Leaf surfaces vary a lot among different species [36]—for example, from the smooth surfaces of amorphous waxes to rough surfaces with fine epicuticular wax crystals. Moreover, the composition of the wax layer is closely related to the leave wettability, which varies among leaf development stages and leaves sides [37].

In agriculture, the evaporation process of a pesticide droplet consists of evaporation during droplet transport and on the leaf surface, without considering atmospheric factors, such as temperature, wind, and humidity. The former process is mainly influenced by the droplet properties, which can be changed if a surfactant is added. Surfactants are widely used to facilitate the wetting of surfaces and spreading of water-based formulations of agrichemicals [38, 39]. Adding surfactants to the aqueous droplet can considerably suppress evaporation, especially for insoluble monolayers in the condensed state [40]. But some surfactants can also facilitate the water evaporation, which decreases the contact angle and thus increases the contact line radius [16, 41–43]. While the latter process is closely related to the droplet properties, surface roughness, surface strain, and so on.

Motivated by these previous studies, herein, the impact of the surfactant concentration on contact angle, contact diameter, droplet height, and evolution of the evaporative droplet volume on rice leaf surfaces was explored in this research. The experimental results were compared to Popov’s model [22]. This study should provide theoretical data concerning the evaporation of the adjuvant and support practical application of pesticide droplets in the future.

## Materials and methods

### Plants and sample preparation

The seeds of the rice variety used in this study are called Dongjin (*Oryza sativa ssp*. japonica). It is commonly cultivated in South China and provided by the Institute of Plant Protection, Chinese Academy of Agricultural Sciences. After seeding on a water bed, the rice plants were transplanted into small plastic pots containing soil. And after three leaf stages, the seedlings were transplanted into oblong plastic pots (50 cm × 30 cm × 17 cm) inundated with water. Three seedlings were sown in one plant pot in the glasshouse under the same controlled environmental conditions: photoperiod, 14 h; relative humidity, 65–80%; and day and night temperatures of 30 and 26°C, respectively. A rice plant with seven leaves was chosen for this experiment.

The fresh rice leaves were fixed to glass slides using a double-sided adhesive tape. The fresh leaves, with the middle vein removed leaving only the side leaf, were adhered to the glass slide. Touching the leaves and placing them in contact with other surfaces were avoided during the experiment to maintain the leaf structure.

### Surfactant

Surfactant was provided by the Dauni Research Center of Advanced Science and Technology Co., Ltd, which consisted of hydrophilic polyoxyethylene moieties, hydrophobic alkyl chains, and a main-chain with positive and negative segments. The chemical structure of the surfactant is poly(acrylic acid/dimethyldiallylammonium chloride/octadecyl methyl-acrylate). The molecular weight of the surfactant is 300000–400000.

Five surfactant solution concentrations (wt.% = 0.001%, 0.005%, 0.01%, 0.05%, and 0.10%) were prepared and diluted with deionized water. For comparison, droplets of pure water were also prepared in these experiments.

### Measurements of the contact angle of droplet and surface tension of surfactant

Measurements of contact angles (CA) were performed with a high-speed optical CA measuring device OCAH200 (precision: ±0.01, Data Physics Instruments GmbH, Filderstadt, Germany) equipped with a high-speed CCD video system and the built-in software SCAT, which could automatically measure static and dynamic CA values.

The droplets were observed during their whole evaporation time and photographed at 1-s time intervals. The high-speed CCD video system computed contact angle, contact diameter, droplet volume (mass), and droplet height at every instant. The droplets were allowed to evaporate in a chamber with a small hole at the top, which was controlled at 27±1°C (same as the temperature in laboratory environment) and a humidity of 26.1%. A droplet with an initial volume of 4 *μ*L was dispensed using a carefully calibrated microsyringe Gastight-1750 Trace syringe (Hamilton Switzerland) through the hole and deposited onto the rice leaf surfaces. It is much smaller than the capillary length (which is 2.7 mm for a water droplet [8]), so the influence of gravity could be neglected. Five concentrated of surfactant solutions and pure water were deposited onto rice leaf surfaces. The experiment was repeated three times to ensure the repeatability.

Surface tension of surfactant was measured by OCAH200 (precision: ±0.01, Data Physics Instruments GmbH, Filderstadt, Germany) and DCAT21 (Data Physics Instruments GmbH, Germany).

## Results and discussions

### The surface tension of the surfactant

**Fig 1:**
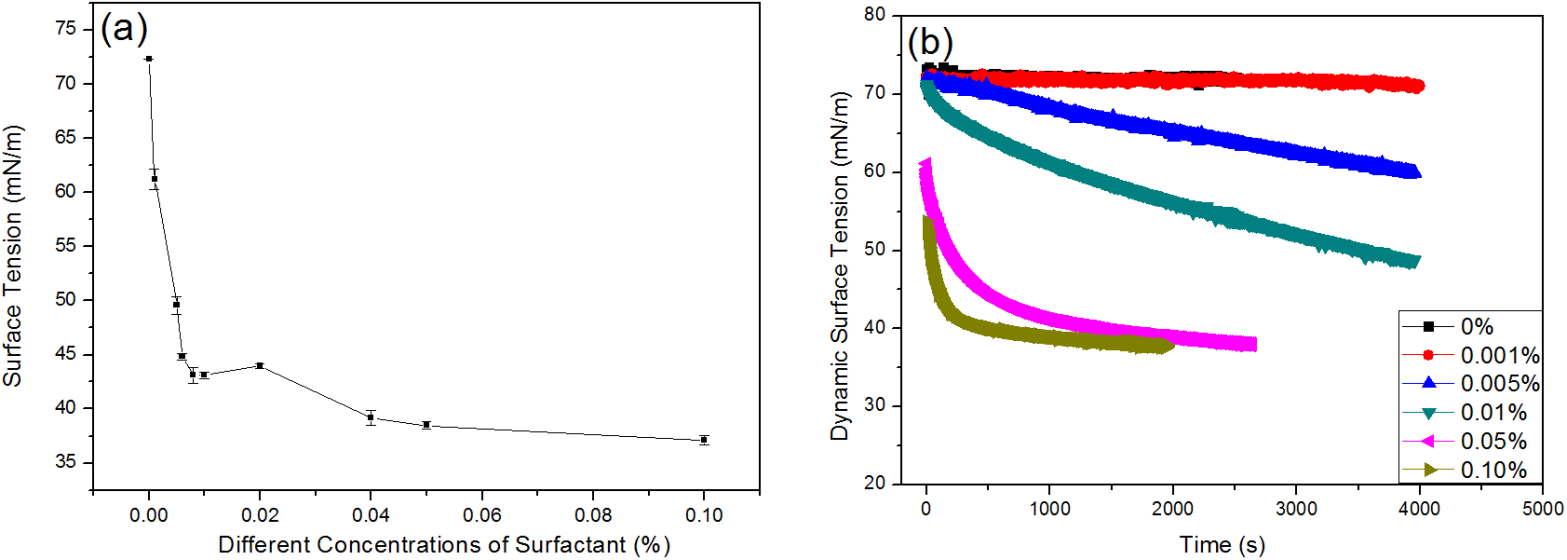
**The surface tension for different concentrations of surfactant solution was measured by the normal Pt**-**plate (a) and the pendant**-**drop method (b).**

Surface tension of the surfactant solution was measured using the normal Pt-plate and the pendant-drop method. As shown in Fig 1A, the critical micelle concentration (CMC) is 0.01%. Fig 1B shows the concentration dependency effect on dynamic surface tension of the surfactant in the long-time regime. When the concentrations are lower than CMC (0.001%, 0.005%), more than 2000 seconds is needed for the dynamic surface tension curves to reach the equilibrium, and such long time will lower the measurement precision on account of the water evaporation. Conversely, when the concentrations were higher than CMC (0.05%, 0.10%), the surface tension takes a short time to reach the equilibrium, and the value is near 37 mN m^-1^ at the concentration of 0.10%. Obviously, the surface tension decreases with the surfactant concentration increase.

When the concentrations become lower than CMC, the surface tension decreases slowly by a slow adsorption of the molecules at the liquid-vapor interface; while the concentration is higher than CMC, the interfacial concentration becomes higher as the time continues, thus leading to a decrease in the surface tension. A fast absorption of molecules in the liquid-vapor interface occurs with the increasing concentration.

### Typical evolution of droplet contact angle and contact diameter on rice leaf surfaces

**Fig 2:**
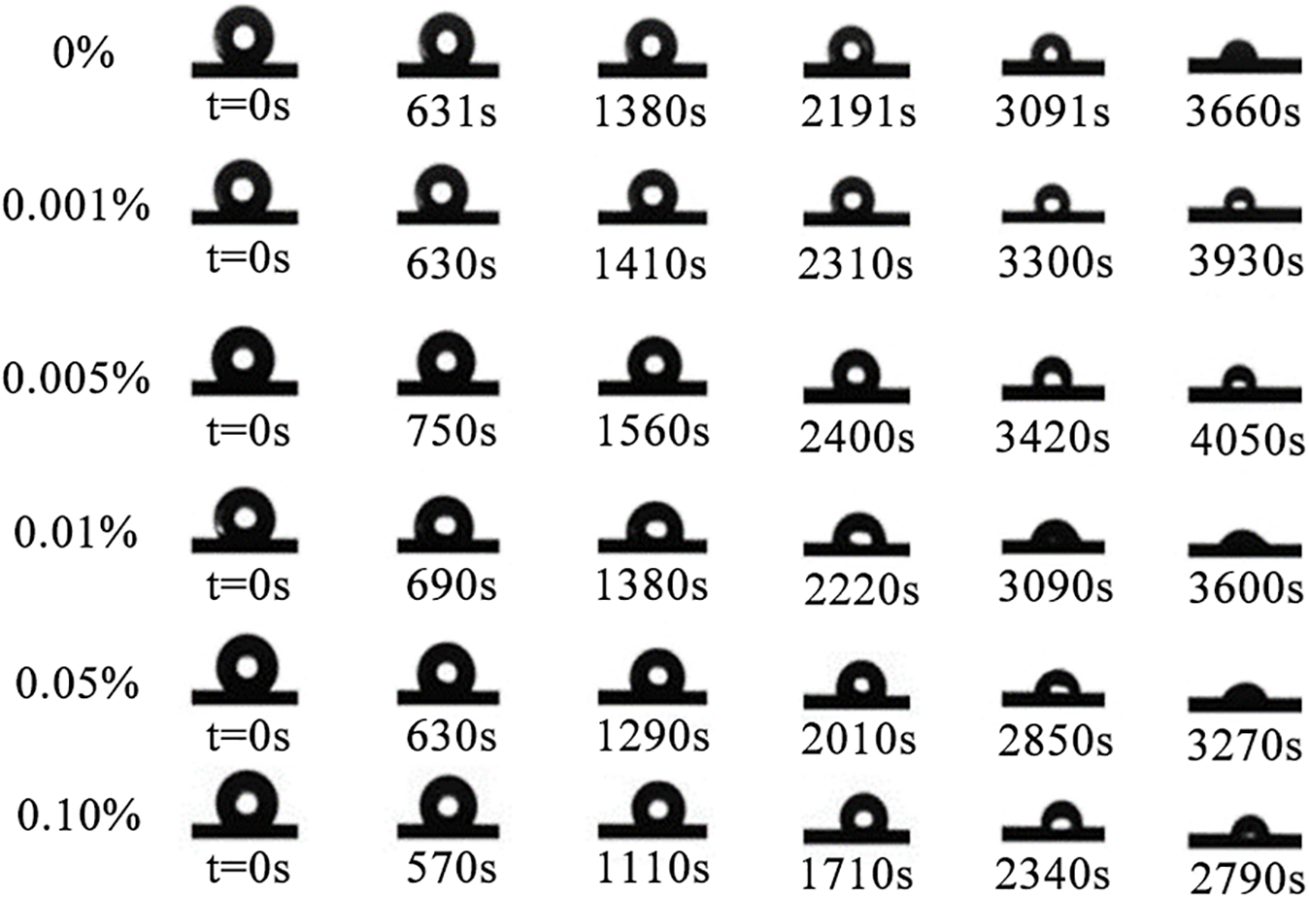
The typical changes in contact angle and contact diameter of pure water droplets (0%), relatively low concentrations (0.001%, 0.005%), and high concentrations (0.01%, 0.05%). Furthermore, various evaporation times to achieve the same normalized volume is listed under each image.

Fig 2 shows the developments in the droplet shapes during evaporation under different the concentrations varying from 0% to 0.001%, 0.005%, 0.01%, 0.05%, and 0.10%, and various evaporation times to achieve the same normalized volume is listed under each image. Compared to droplet of pure water (0%), the shapes of droplets containing lower concentrations of surfactant change a little over the whole evaporation period. With the concentration increase from 0.01% to 0.05%, the leaf wetting by the droplets is improved and a CCR mode is nearly observed, which is primarily influenced by the surfactant and the hydrophobicity of the leaf surfaces.

### Droplet evaporation kinetics on rice leaf surfaces

**Fig 3:**
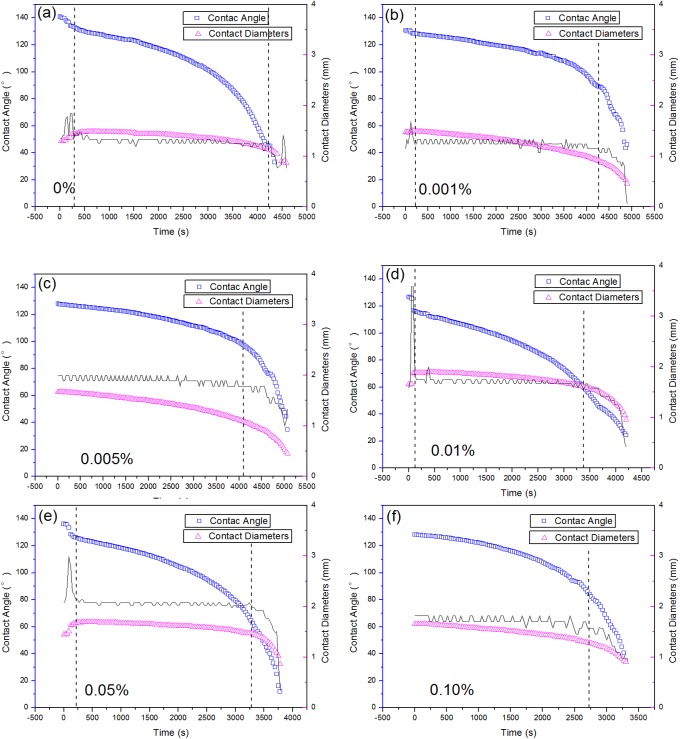
**Evolution of the contact angles (blue squares) and contact diameters (red triangles) of a sessile droplet of (a) pure water droplets and (b–f) droplets containing different concentrations of surfactant over the evaporation period on rice leaf surfaces.** The distinct evaporation periods (the steady start stage, CCR, and mixed mode) are indicated by vertical dotted lines. The vertical lines are determined by the significant change in the slope of the contact diameter shown by black lines in the picture.

Fig 3 shows the evolution of the contact angle and contact diameter of a sessile droplet under different concentrations of surfactant solutions during evaporation on rice leaf surfaces. The evaporation of droplets containing 0.001% (Fig 3B), 0.005% (Fig 3C), and 0.10% (Fig 3F) are dominated by a mixed mode of evaporation (decreasing contact angle and contact diameter). The distinct evaporation periods (in the mixed mode) are indicated by vertical dotted lines. A more rapid decrease in contact angle and a quicker evaporation of the droplet could be observed for a concentration of 0.1%, which is the main difference between 0.001%, 0.005%, and 0.10%. In contrast, the droplets of other surfactant concentrations undergo three sequential evaporation periods (start steady stage → CCR mode → mixed mode), as shown in Fig 3A (pure water), Fig 3D (0.01%), and Fig 3E (0.05%), respectively. In addition, the distinct evaporation periods are indicated by vertical dotted lines.

Notably, the mechanism of aqueous surfactant solutions spreading over hydrophobic substrates have not yet been completely understood [39], since the spreading dynamic of surfactant solutions is affected by the following: (i) a time-dependent adsorption/desorption of surfactant molecules at all interfaces (i.e., liquid/vapor, solid/liquid, and solid/vapor) is involved, which can drastically change the interfacial tension and the energy balance at the moving three phase contact line; (ii) resulting interfacial tension gradients and Marangoni flow as a consequence; and a (iii) disjoining/conjoining (Derjaguin's) pressure gradient. At the concentrations of 0.001% and 0.005% (C<CMC), droplets show a slower decrease in contact diameter and contact angle than others. As reflected in Fig 1B, molecules transported to the three-phase contact line is delayed by a slow absorption at the liquid-vapor interface. Therefore, a worsened wetting state occurs on the hydrophobic rice leaf surfaces, which keep the droplet in the Cassie-Baxter state. In this case, the contact between the liquid with the leaf surface is minimal; therefore, air is contained among the three-phase contact line which can facilitate the evaporation of the droplet and result in the existence of the narrow wedge region. Within this region, the diffusion of liquid vapor is highly restricted during evaporation [32]. Besides, the evaporation of the mixed mode results in a little change of the droplet shape, which prolongs the presence time of the narrow wedge region. It is assumed that the narrow wedge region disappears at a contact angle of ~ 95°.

On the other hand, better wetting of the rice leaf surfaces occurs with a decrease of surface tension for the solutions containing 0.01% and 0.05% surfactant (Fig 3D and 3E), and then results in a lower air content at the three-phase contact line, consequently suppressing the evaporation of droplet at the three-phase contact line. The trend of a transition from the Cassie-Baxter to the Wenzel wetting state is characterized by a sudden decrease in contact angle and increase in contact diameter at the beginning steady stage. In this case, the evaporation kinetics shows an almost constant contact diameter and a decreasing contact angle (CCR mode). In addition, the unchanged contact diameter indicates strong contact line pinning [44] throughout the evaporation process. It is proposed that the strong contact line pinning is induced by the absorption of surfactant. This kinetic evaporation progress causes a rapid change to the shape of the droplet, resulting in a quicker disappearance of the narrow wedge region and a reduction in the evaporation time. The pinning of the droplet boundary is related to the intermolecular interactions at the three-phase contact line [45, 46] and is affected by the surface morphology, chemical heterogeneity, and interfacial wetting states [47–49].

As shown in Fig 3F, corresponding to 0.10% droplet, the highest concentrations of surfactant in this experiment underwent evaporation via the mixed mode, which results in a sharp decrease in contact angle, thus leading to a significant reduction of the narrow wedge region. Aside from the effect of the narrow wedge region, the molecules of the high concentrations of adjuvant may occupy too much space. Water in the droplet is squeezed out to the surface, resulting in an increase in the rate of evaporation. Furthermore, a sharp decrease of contact angle and contact diameter occurs under all concentrations at the end of the evaporation when less water and higher concentrations of surfactant are present.

**Fig 4:**
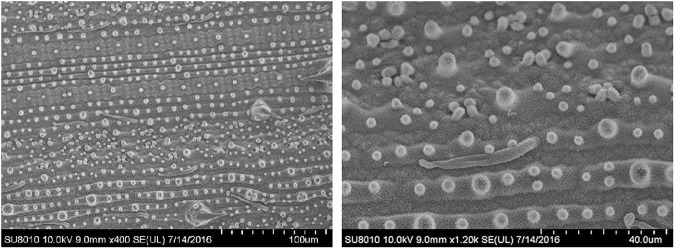
The scanning electron micrograph of the rice leaf surface used in the experiment.

These evaporation periods depend on the surfactant concentrations and the hydrophobicity of the rice leaf surface. Scanning electron micrograph (SEM) of the rice leaf surface is shown in Fig 4. Apparently, it is hard for droplets with lower concentrations to make a smooth wetting state on this disordered superhydrophobic leaf surface.

**Fig 5:**
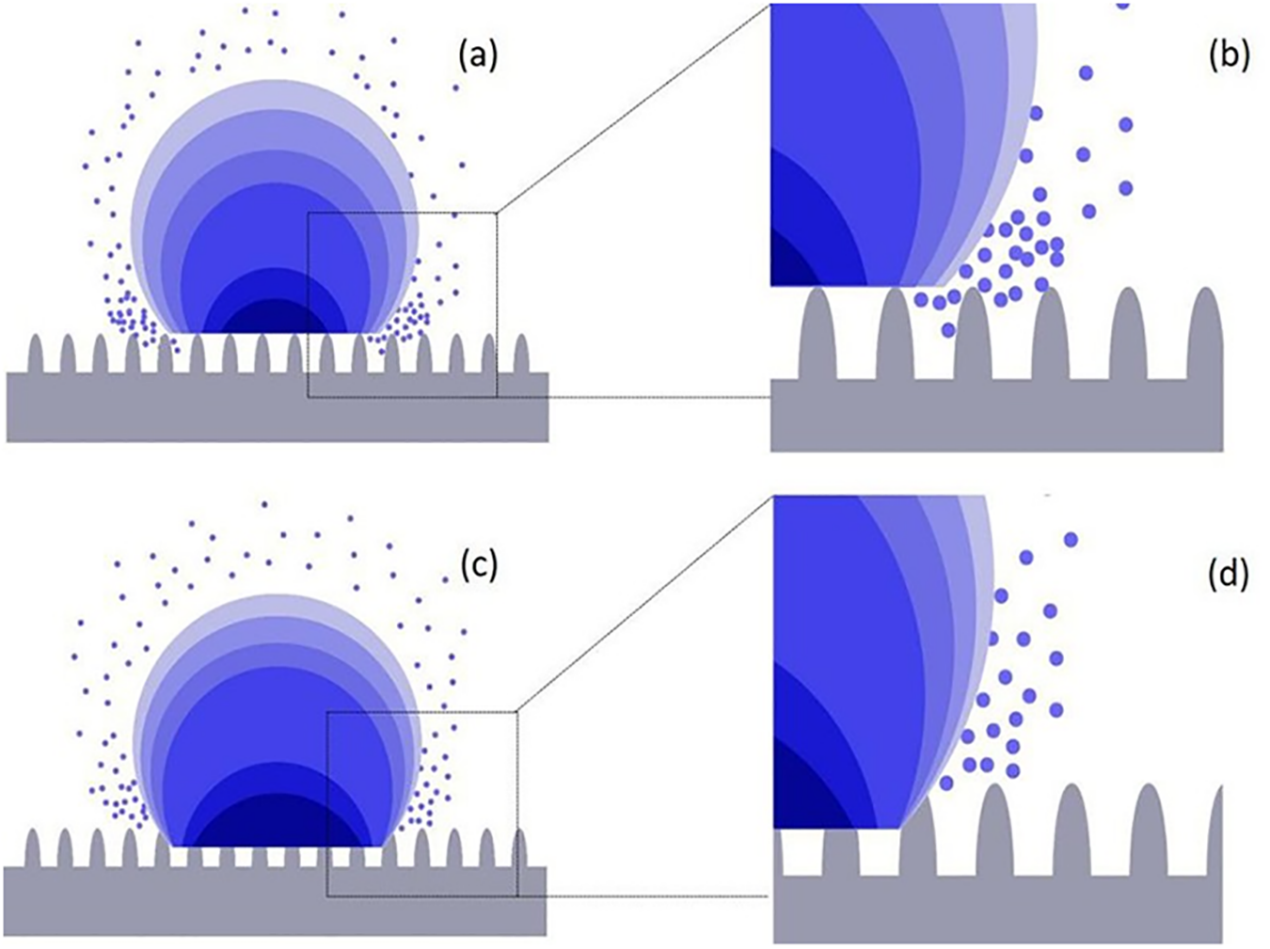
Sketches of two main droplet evaporation mode for droplets containing low concentrations of (a, b) 0.001% to 0.005% and high concentrations of (c, d) 0.01% to 0.05%. **Figs (b) and (d) show enlarged sections of (a) and (c), respectively.** The color changes are used to represent the evaporation of the droplets. The blue dots respect the vapor evaporated from the droplet surface. Also, the density in Fig (a) with a worse wetting state is higher than that of Fig (c), which has a better wetting state. The higher density of vapor within the small narrow region also restricts the evaporation rate of the droplet.

To further clarify the evaporation behavior, the evaporation trends of droplets under different concentrations of surfactant are shown in Fig 5A and 5B (low concentrations of 0.001% and 0.005%) and in Fig 5C and 5D (high concentrations of 0.01% and 0.05%). As shown in Fig 5A for the concentrations of 0.001% and 0.005%, the poor wetting state results in a large amount of air content at the three-phase contact line, facilitating the evaporation of water and contributing to a decrease in contact angle and contact diameter. This evaporation does not change the profile of the droplet, but may result in a longer evaporation duration of the very narrow wedge region magnified in Fig 5B, where the diffusion of liquid vapor is restricted during evaporation [32]. In contrast, for the concentrations of 0.01% and 0.05%, the air content is lower at the three-phase contact line, which results in a better wetting state on the surface of the rice leaves, as is shown in Fig 5D. Such behavior is consistent with the CCR mode (i.e., constant contact diameter and decreasing contact angle) during the majority of the evaporation phase, followed by a mixed mode (i.e., decrease in both contact angle and contact diameter) near the end. This evaporation type results in a rapid change to the shape of the droplet and a reduction in the evaporation time during which a narrow wedge region appears.

**Fig 6:**
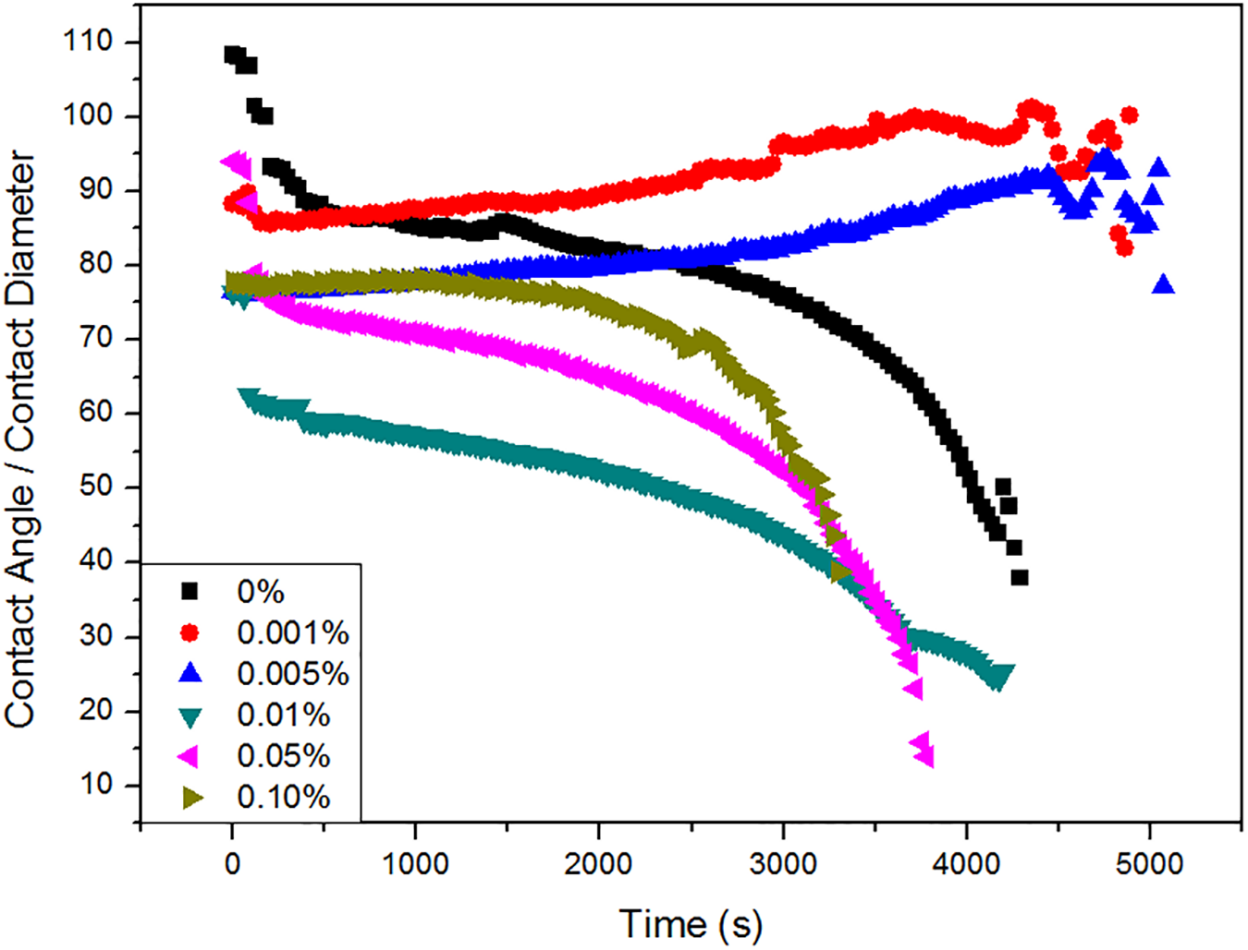
Evolution of the ratio of contact angle and contact diameter (contact angle/contact diameter vs. time) for pure water droplets and droplets containing different concentrations of surfactant over the evaporation period.

As shown in Fig 6, the contact angle is divided by the contact diameter. After the start of the steady stage, the ratio exhibits two different trends. A decreasing ratio signifies a more rapid change in contact angle than in contact diameter. However, the increasing ratio indicates a more rapid change in contact diameter than contact angle. Besides, the increasing ratio for droplets with concentrations of 0.001% and 0.005% is beneficial in maintaining the droplet profile, which prolongs the presence of the narrow wedge region. Moreover, a decrease in the ratio indicates a faster changing contact angle, leading to a more rapid disappearance of the narrow wedge region, which is consistent with the conclusions from the data in Fig 3.

### Droplet volume and droplet height on rice leaf surfaces

**Fig 7:**
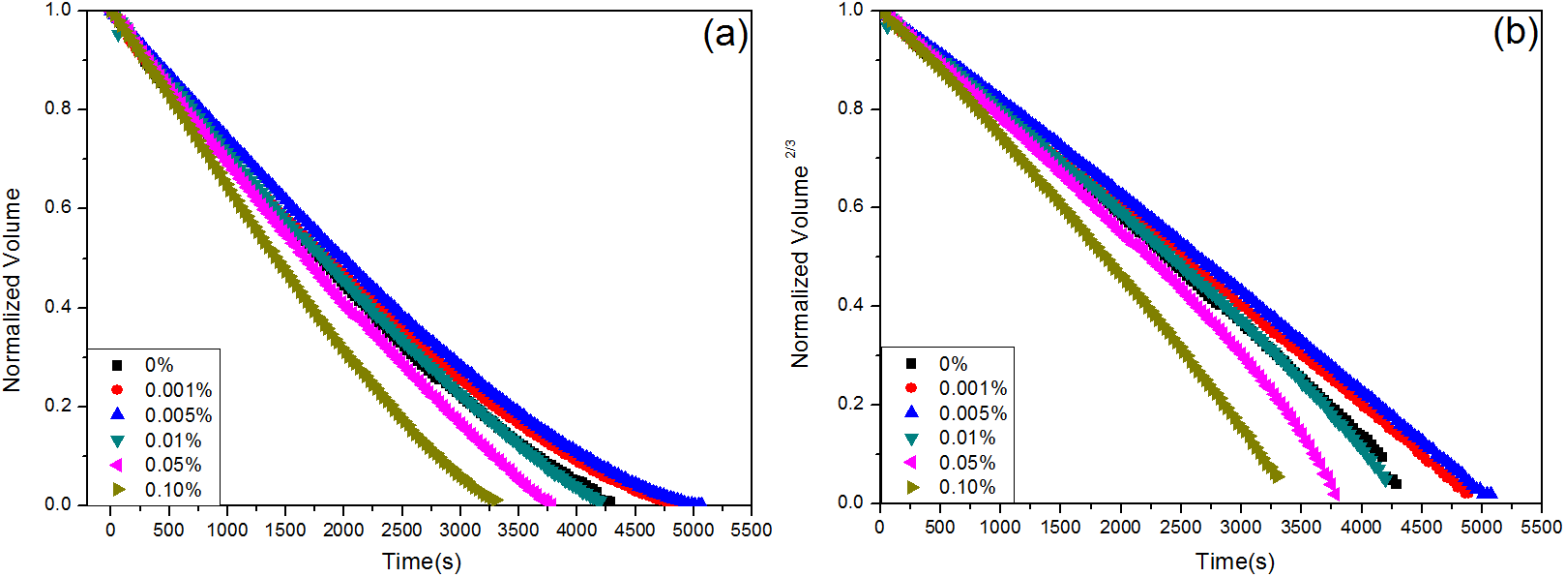
(a, b) Evolution of the volume of a pure water droplet and droplets containing different concentrations of surfactant over the whole evaporation time, (*v*/*v*_0_) vs. *t* and (*v*/*v*_0_)^2/3^ vs. *t*, respectively.

**Fig 8:**
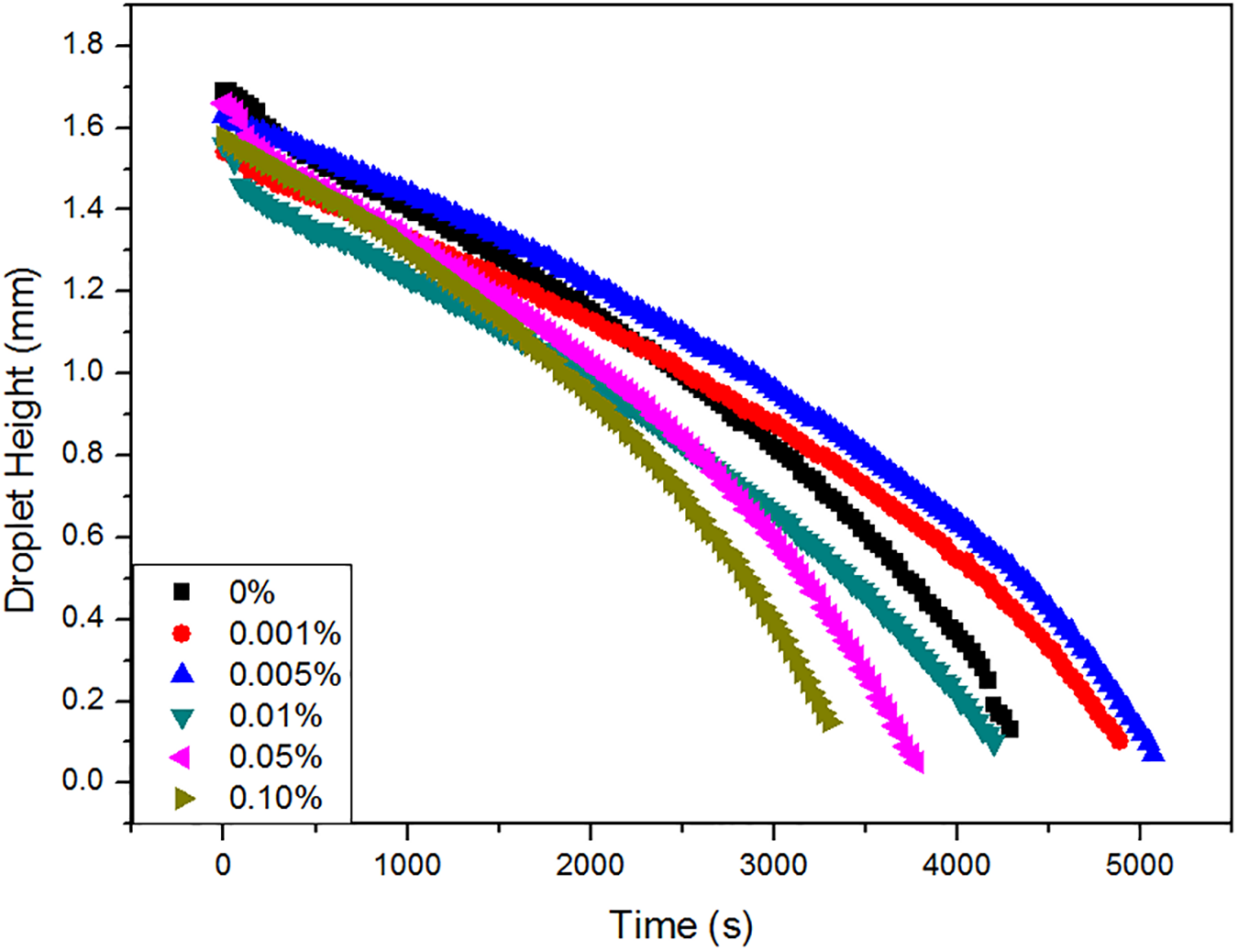
Evolution of the height for water-only droplet and droplets of different concentrations of surfactant with an initial volume of 4 μL on the surface of a rice leaf.

Fig 7A shows the evolution of the droplet volume with evaporation time (*v*/*v*_0_ vs. *t*). The plot is initially linear, and then deviates from linearity at the end of the droplet lifetime. In addition, the evaporation time of the droplet volume is related to the surfactant concentration: (1) when the surfactant concentrations are 0.001% and 0.005%, the evaporation time is longer than that of the water droplet; (2) similar evaporation times are observed for droplets containing 0.01% surfactant concentrations and pure water; (3) when the surfactant concentrations of droplets are 0.05% and 0.10%, the evaporation time is reduced, and such trend is more significant at higher concentration levels. The deviation from linearity in Fig 7A is assumed to be due to the existence of narrow wedge regions in water, droplets containing 0.001% and 0.005% surfactant concentrations, as well as the better wetting state of droplets with concentrations of 0.01%, 0.05%, and 0.10%. But a stronger linear relationship is obtained by using the power-law model [(*v*/*v*_0_)2/3 vs. *t*)] as shown in Fig 7B, and this situation allows a more accurate prediction of the volume transfer rate than the initial model (*v*/*v*_0_ vs. *t*). Furthermore, the gradients of fitted lines are -1.99, -1.86, -2.05, -2.26, and -2.68 (10^−4^/s) for surfactant concentrations from 0.001% to 0.10%, respectively, whereas that of a pure water droplet is only -2.07 (10^−4^/s). The more rapid evaporation rate indicates the more rapid evaporation of the droplet, which is consistent with the data shown in Fig 7A. Moreover, the evolution of the droplet height was also investigated as shown in Fig 8. The evaporation rate can be reflected by the change of droplet height [10], and the droplet heights for all droplets decrease with increasing evaporation time. Although the rates changed in a different way, it has a similar trend to that obtained for volume changes. Different concentrated droplets have nearly the same height. It is obvious that the droplet height changed slower than that of water under the concentrations of 0.001% and 0.005%, while it changed faster than that of water with increasing concentrations from 0.01% to 0.05%.

**Fig 9:**
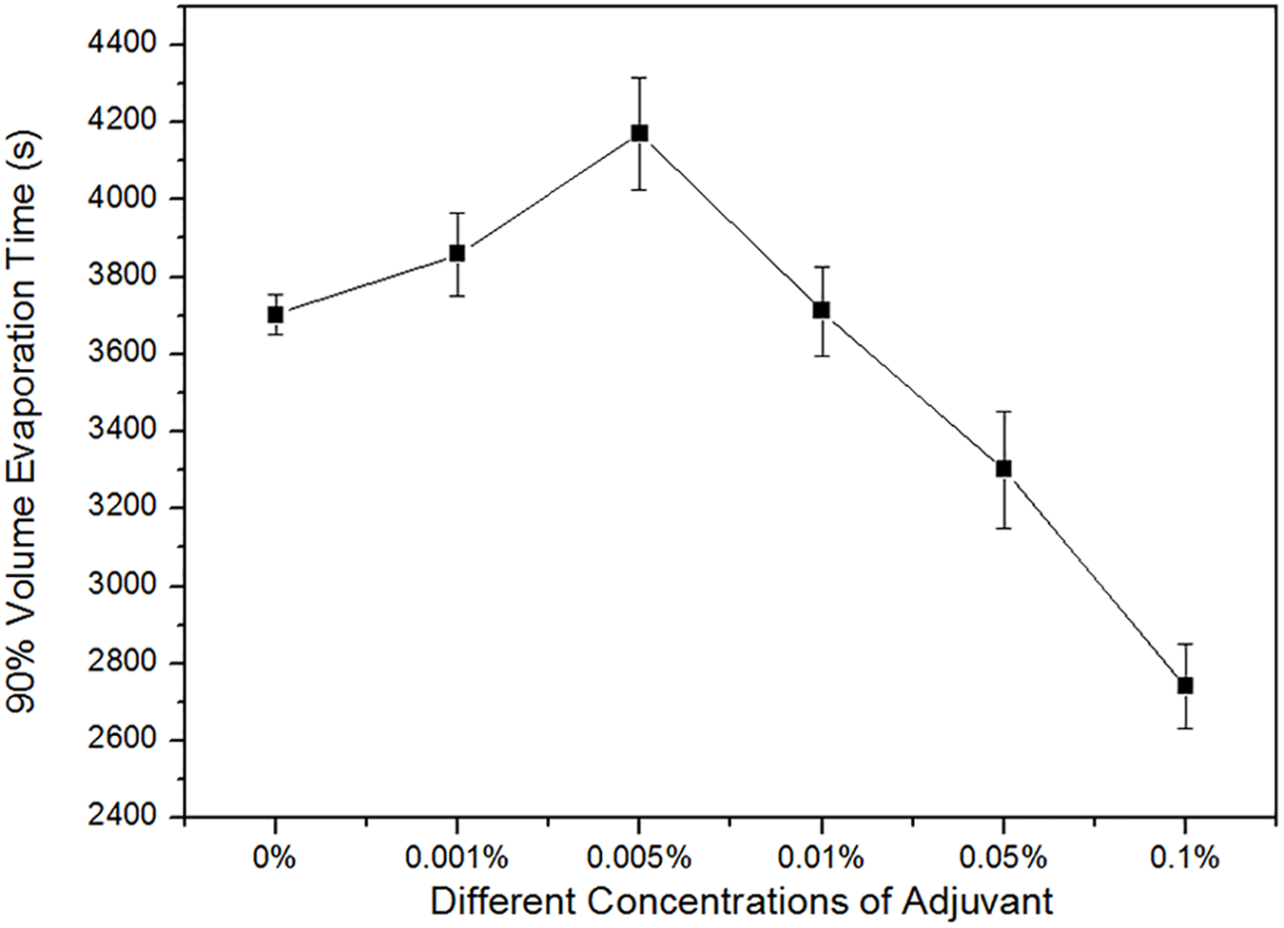
Average time required to evaporate 90% of the volume of a pure water droplet and those containing different concentrations of adjuvant (initial volume ≈ 4 μL) on natural rice leaf surfaces.

In addition, the evaporation time required for the first 90% droplet volume (∼4 μL) (avoiding the measurement errors when the contact angle is too small to be calculated accurately) of water and different concentrated surfactant on rice leaf surfaces is shown in Fig 9. The evaporation time gradually increased with the increase in surfactant concentration, i.e., from water to the concentration level of 0.005%. Furthermore, the evaporation time is significantly reduced, even lower than that of water containing relatively high concentrations from 0.01% to 0.1%. That is, relatively the low surfactant concentration can prolong the droplet evaporation time, while higher surfactant concentrations correspond to the quicker droplet evaporation. As with to the volume change, this behavior can be attributed to the existence of a narrow wedge region at relatively low surfactant concentrations and a better wetting state at higher concentrations, i.e., 0.01% to 0.10%.

### Comparison between theory and experiment

Based on the Popov’s model [22], Hanneke Gelderblom *et al*. [23] investigated the evaporation of water droplets on a superhydrophobic substrate. To describe a universal behavior that is independent of drop size and other parameters *c*_*s*_, *H*, *ρ*, and *D*, they introduce the nondimensional mass and time as

$$\hat{M}=\frac{M}{\rho R^{3}}\;\hat{t}=\frac{c_{S}-c_{\infty }}{\rho }\frac{t}{R^{2}/D}$$ (1)

where *M* is the mass of the droplet, *t* is the evaporation time, *ρ* is the density of the liquid (approximately to the density of water for the low concentrations of surfactant and the evaporation of water vapor), *R* is the contact radius of the droplet, *D* is the diffusion coefficient, *c*_s_ and *c*_*∞*_ are the saturated vapor concentration at the droplet surface and far from it respectively. Then the relations that only rely on the contact angle can be obtained:

$$\frac{d\hat{M}}{d\hat{t}}=-\pi f(\theta )$$ (2)

$$\hat{M}=\pi \frac{\cos^{3}\theta -3\cos\theta +2}{3\sin^{3}\theta }$$ (3)

$$\frac{d\theta }{d\hat{t}}=-{\left(1+\cos\theta \right)}^{2}f(\theta )$$ (4)

In these formulas, *θ* is the contact angle and *f(θ)* is the functional variation of contact angle evaluated using a numerical integration scheme in MATLAB[50].

$$f\left(\theta \right)=\frac{\sin\theta }{1+\cos\theta }+4\int_{0}^{\infty }\frac{1+\cosh\;2\theta \tau }{\sinh\;2\pi \tau }\tanh[(\pi -\theta )\tau ]d\tau$$ (5)

In our experiment, we used the following conditions: temperature of 27±1°C, *D* = 22.5×10 ^−6^ m 2 /s, *ρ*∼ 996.5 kg/m^3^, *c*_*s*_ = 2.60 × 10 ^−2^ kg/m^3^, and *c*_*∞*_ = *H c*_*s*_ (all obtained from [51]) by linear interpolation).

**Fig 10:**
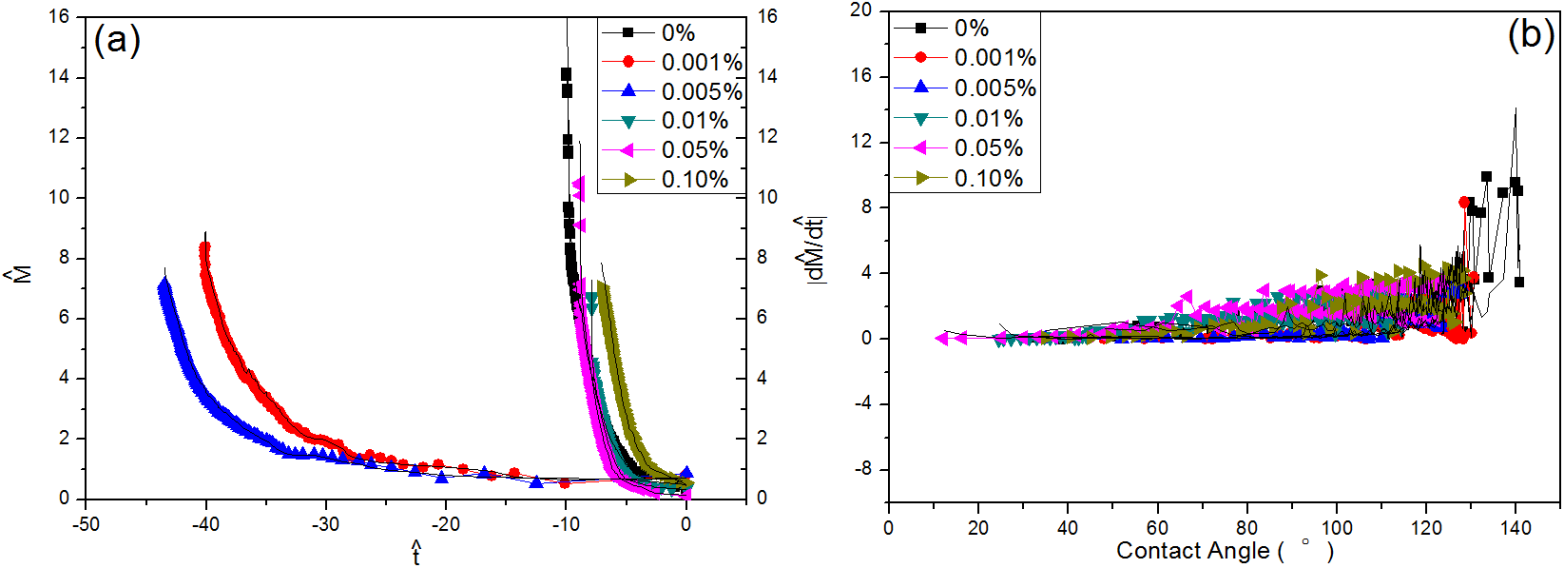
**The dimensionless droplet mass plotted against the dimensionless time (a). The rate of mass loss of the droplet vs the contact angle (b).** The color lines represent the experimental data, which are derived from the measured droplet volume. The black solid lines represent the theoretical prediction according to Popov’s model. The experimental data are scaled according to (6). The time is set to 0 at the end of the droplet life.

To make a comparison between theory and experiment, the experimental data is rescaled according to (1). The time is set to 0 in the end of the droplet life. Meanwhile, the droplet volume, contact angle, and contact radius can be obtained independently. Then, relation (3) is used to derive the droplet mass theoretically according to the change of contact angle. Furthermore, the rate of the mass loss given the contact angle can be obtained by Eqs (2) and (4) to make a comparison between the experiment and theory. Fig 10A shows the dimensionless droplet mass plotted against the dimensionless time. The color lines represent the experimental data, which are derived from the measured droplet volume. The black solid lines represent the theoretical prediction according to Popov’s model [22]. Clearly, the experimental data is in excellent agreement with the theoretical prediction (3). Fig 10B exhibits the rate of mass loss of the droplet vs. the contact angle. Basically, the experimental date can roughly collapse onto the theoretical curves for the superhydrophobic rice leaf surface according to the “diffusion-limited” Popov model. The disordered microstructure structure is shown in Fig 4, and the chemical heterogeneity of leaves and the presence of the narrow wedge region can be seen in Fig 3.

Fig 10A shows the dimensionless droplet mass plotted against the dimensionless time. Fig 10B shows the rate of mass loss of the droplet plotted with the contact angle. The color lines represent the experimental data, which are derived from the measured droplet volume. The black solid lines represent the theoretical prediction according to the Popov model. The experimental data are scaled according to (6). The time is set to 0 at the end of the droplet life.

## Conclusions

Evaporation of the water droplets containing different concentrated surfactants on fresh rice leaf surfaces are shown to occur by a mixed mode for droplets with concentrations of 0.001% and 0.005%, whereas both a CCR and a mixed mode occur in water droplets and those with concentrations of 0.01%, 0.05%, and 0.10%. The experimental results and analysis reveal that the assumptions of the “diffusion-limited” model are approximately applicable in the case of droplets of water and different concentrated surfactant solutions on rice leaf surfaces. The evaporation kinetics of the droplet is closely related to the surfactant concentrations and the characteristics of rice leaf surfaces. It is found that when the concentrations of droplets are 0.001% and 0.005%, the adsorption of the molecules at the liquid-vapor interface is too slow to make a good wetting state for the droplet. The evaporation of the droplet is mainly in a mixed mode, which results in the long existence of a narrow wedge region due to the slow change in the droplet profile. Furthermore, the presence of the narrow wedge region restricts the diffusion of liquid vapor, which contributes to the longer evaporation time compared to water without any surfactant. However, due to the improved wetting state in higher levels of concentration such as 0.01% and 0.05%, the mainly decrease of contact angle in the CCR mode results in a quicker disappearance of the narrow wedge, which then reduced the evaporation time. Furthermore, the evaporation time is significantly reduced in droplets containing 0.10% surfactant, partly because a large proportion of the droplet volume is occupied by the surfactant molecules. This result is further confirmed by the time required for the loss of the first 90% droplet volume. The same trends are observed for the evolution of droplet volumes and droplet heights. The results proved to a roughly collapse to the theoretical curves based on the model presented by Popov [22].

In the field of agriculture, the addition of a surfactant could effectively enhance the wetting, retention, and retention of pesticide droplets on target crops [52]. Consequently, the evaporation of droplets containing surfactant is of great significance for the effective utilization of surfactants and pesticide [53, 54] retention. In this work, the evaporation of droplets containing relatively high surfactant concentrations of 0.01% and 0.05% were dominated by the CCR mode, which shows a decreasing contact angle and almost constant diameter. It is beneficial for the droplet to obtain a steady state on rice leaf surfaces and to further prevent the droplet from rolling off from the leaf surface. In addition, the improved wetting and a reduced evaporation time gives longer retention times of the surfactant and helps to reduce droplet drift. Therefore, our study of the evaporation of surfactant solutions on rice leaf surfaces is important to understand the effect of surfactant addition in water and guide the utilization of surfactants and pesticides in agriculture.
